# Molecular Pathway and Regulatory Mechanism of the Saponin Biosynthesis in Sea Cucumber *Apostichopus japonicus*

**DOI:** 10.3390/md24070230

**Published:** 2026-06-30

**Authors:** Pingzhe Jiang, Shan Gao, Yujun Liu, Zhong Chen, Liang Zhao, Zelong Zhao, Feifei Zhang, Yongjia Pan, Yao Xiao, Guohan Zhang, Jingwei Jiang, Zunchun Zhou

**Affiliations:** 1 Key Laboratory of Protection and Utilization of Aquatic Germplasm Resource, Ministry of Agriculture and Rural Affairs, Dalian 116023, China; jiangpingzhe@163.com (P.J.); gs_7920@163.com (S.G.); lyj7005@126.com (Y.L.); ch_zhong@163.com (Z.C.); ouczhaoliang@126.com (L.Z.); zzl2516@163.com (Z.Z.); z_20201109@163.com (F.Z.); pyjsky866@163.com (Y.P.); m17604117050@163.com (Y.X.); lnshky_zgh@163.com (G.Z.); 2 Key Laboratory of Germplasm Improvement and Fine Seed Breeding for Marine Aquatic Animals, Dalian 116023, China; 3 Liaoning Ocean and Fisheries Science Research Institute, Dalian 116023, China

**Keywords:** sea cucumber *Apostichopus japonicus*, marine saponin, biosynthesis, key enzyme, regulatory mechanism

## Abstract

Sea cucumber *Apostichopus japonicus* is one of the few animals capable of synthesizing saponins, which are critical components of its nutritional quality and health-beneficial properties. However, the specific mechanism underlying saponin biosynthesis in sea cucumbers remains unclear despite previous investigations. This study aimed to characterize the molecular pathway and regulatory mechanism of saponin biosynthesis in *A. japonicus*. Thirteen candidate genes involved in de novo saponin skeleton synthesis were identified from the *A. japonicus* genome, and their full-length cDNAs were obtained via PCR-RACE. Sequence analysis predicted the intracellular localization of these genes. Combined in situ hybridization and quantitative real-time PCR analyses revealed their high expression in coelomocytes, indicating coelomocytes as the primary saponin synthesis sites. Knockdown of mevalonate kinase (AjMVK) and two oxidosqualene cyclases (AjPS and AjLS) caused a more obvious decrease in saponin levels, identifying them as key biosynthetic enzymes. Yeast two-hybrid assays revealed that AjPS and AjLS interact with ficolins, complement component 3-2, O-linked β-N-acetylglucosamine transferase, and α-L-fucosidase, whose regulatory effects were further validated by RNA interference and saponin content measurements. These results suggest that saponin biosynthesis in *A. japonicus* is regulated by the complement lectin pathway and modulated by glycosylation enzymes, providing a molecular foundation for enhancing bioactive saponin production for pharmaceutical and nutraceutical applications.

## 1. Introduction

The sea cucumber *Apostichopus japonicus* is an aquatic species with the highest single-species output value. The high economic value of *A. japonicus* stems from its high nutritional, medicinal, and health care value. Since ancient times, *A. japonicus* has been known as the “ginseng of the sea” [1,2]. Several studies have identified saponins, which are absent in most other animals, as the active substances in *A. japonicus*, endowing sea cucumbers with their multiple medicinal and health care activities. Numerous studies have demonstrated that sea cucumber saponins exhibit a broad spectrum of biological activities, including antioxidant [3], antidiabetic [4], antitumor [5], immunomodulatory [6], and antifungal [7] effects. These biological properties endow sea cucumber saponins with promising application prospects in pharmaceutical development, functional food and nutraceutical industries. As the main bioactive components of *A. japonicus*, saponins are predominantly accumulated in the body wall, which is the most widely used tissue for saponin extraction and quantitative determination. Therefore, clarifying the molecular basis of sea cucumber saponin biosynthesis can not only deepen our understanding of marine invertebrate secondary metabolism but also provide theoretical support for efficient exploitation and utilization of these natural bioactive compounds.

As secondary metabolites, saponins are widespread in plants but have only been found in a few marine animal species, such as sea cucumbers, starfish, and sponges [8,9,10,11]. While the saponin biosynthetic pathway has been extensively characterized in terrestrial plants, our understanding of this process in animals remains limited. Thus, the widely documented plant saponin biosynthesis provides an essential reference for investigating the corresponding pathway in *A. japonicus*. According to the literature on plants, the biosynthesis of saponins can be generally classified into three phases. The first phase comprises biosynthesis of isopentenyl pyrophosphate (IPP) together with dimethylallyl pyrophosphate (DMAPP). IPP and DMAPP, which act as precursors for saponin synthesis, are produced via the mevalonate (MVA) pathway, with acetyl-CoA serving as the initial substrate [12]. The second phase consists of 2,3-oxidosqualene synthesis. IPP and DMPP are catalyzed by isopentenyl transferase and cyclase, including farnesyl pyrophosphate synthase (FPS), squalene synthetase (SS) and squalene epoxidase (SE), to produce 2,3-oxidosaqualene. This second stage is characterized primarily by the synthesis of the saponin skeleton. The third phase comprises cyclization, hydroxylation, and glycosylation modifications of 2,3-oxidized squalene. The third stage involves the most complex enzymatic reactions. As a substrate for oxidosqualene cyclases (OSCs) to catalyze its cyclization, 2,3-oxidosqualene is the common biosynthetic precursor of both sterols and triterpenes. When cyclized by cycloartenol synthase or cucurbitadienol synthase, 2,3-oxidosqualene is catalyzed to produce sterols [13]. 2,3-oxidosqualene is also cyclized by β-amyrin synthase, α-amyrin synthase, dammarenediol synthase, or parkeol synthase to produce triterpenes [14,15]. The conversion of cyclic products into final saponins requires a reaction process involving multiple enzymes, such as cytochrome P450 and glycosyltransferases, which are required in ginseng saponin biosynthesis [16].

Previous omics analyses, including genomics and transcriptomics, have revealed that sea cucumbers possess a molecular basis for de novo saponin synthesis similar to that in plants. For example, Mitu et al. elucidated a saponin biosynthesis pathway in *Holothuria scabra* through comparative transcriptome analysis [11]. Liu et al. confirmed that triterpenoid biosynthesis in *Stichopus horrens* proceeds via the MVA pathway. Additionally, Liu et al. uncovered several genes putatively encoding cytochrome P450 and glycosylation enzymes, which may contribute to the post-stage biosynthesis of triterpenoid glycosides [17]. Yang et al. screened 30 genes related to upstream and downstream regions of the saponin biosynthetic pathway, including putative enzyme-encoding genes [18]. Of note, gene functions identified through bioinformatics analysis may exhibit discrepancies, affecting the accuracy of the putative molecular mechanisms underlying saponin synthesis in sea cucumbers. For example, Li et al. identified two OSCs, termed LAS1 and LAS2, in *A. japonicus* via genome sequencing data [19]. In contrast to animal lanosterol synthase (LAS), which converts 2,3-oxidosqualene to lanosterol via cyclization, both LAS1 and LAS2 failed to generate lanosterol and instead yield parkeol and 9β-lanosta-7,24-dienol, respectively [19]. Parkeol has been identified as a precursor for sea cucumber saponins [20], whereas 9β-lanosta-7,24-dienol has been proposed to be a putative precursor, with further validation pending. To further elucidate the molecular pathways of saponin synthesis in sea cucumbers, it is necessary to validate the roles of the candidate genes for saponin biosynthesis identified through omics approaches. Additionally, although multiple genes are involved in saponin synthesis, different genes play distinct roles. Several genes exert dominant regulatory effects on saponin biosynthesis and are identified as candidate key genes of the pathway. Regarding the molecular pathway, the key genes involved in sea cucumber saponin biosynthesis remain unknown.

In addition to providing various biological activities for the organisms in health care, saponins also function as critical immune defense substances for organisms. In plants, saponins significantly contribute to defense systems against pathogens. For example, saponins derived from plant species, including oats, alfalfa, *Solanum* spp., garlic, and *Capsicum annuum* seeds, have bacterial inhibitory activity [21]. For decades, the antifungal properties of plant saponins have been acknowledged, with their principal mechanism against fungi involving pore formation and impairment of membrane integrity [22]. In addition, saponins directly affect the reproduction and growth of pests, thereby enabling plants to resist insects [23]. Similarly, saponins play a role in providing chemical defense against potential assailants as part of the innate immunity of sea cucumbers [4,24]. Once under stress, some sea cucumbers entangle and immobilize invaders through cuvierian tubules as a primary defense, and saponins in the tubules cause subsequent death of the invaders [25]. Saponins in sea cucumbers were reported to participate in immune responses against environmental stress by inhibiting the growth of pathogens and regulating immune-related functions through enhancing natural killer cell activity by stimulating cytokine production, with increased secretion under stressful conditions [26,27]. Although the important roles of saponins in immune defense are relatively clear, it remains unknown how saponin biosynthesis is regulated by immune processes.

Taken together, while genomic and transcriptomic studies have proposed a suite of candidate genes associated with saponin biosynthesis in sea cucumber, systematic in vivo functional validation of these genes is still lacking, and their relative contributions to saponin biosynthesis remain uncharacterized. Additionally, the primary tissue responsible for saponin production in *A. japonicus*, a Cuvierian tubule-lacking species, has not been firmly established, and the regulatory relationship between immune signaling and saponin biosynthesis remains largely unexplored.

Therefore, the present study aimed to elucidate the molecular mechanism of *A. japonicus* saponin biosynthesis from acetyl coenzyme A to the cyclization of 2,3-oxidosqualene, to determine the main synthetic tissues of *A. japonicus* saponins, to identify the key enzymes of the saponin synthesis pathway, and to investigate the potential regulatory effects of immune and physiological factors on saponin biosynthesis. The present results help elucidate the molecular pathway and regulatory mechanism of saponin biosynthesis in *A. japonicus*, and they lay an important foundation for the development and utilization of *A. japonicus* saponin sources.

## 2. Results

### 2.1. Identification of the Molecular Pathway for De Novo Synthesis of A. japonicus Saponin Skeleton Structure

**Figure 1:**
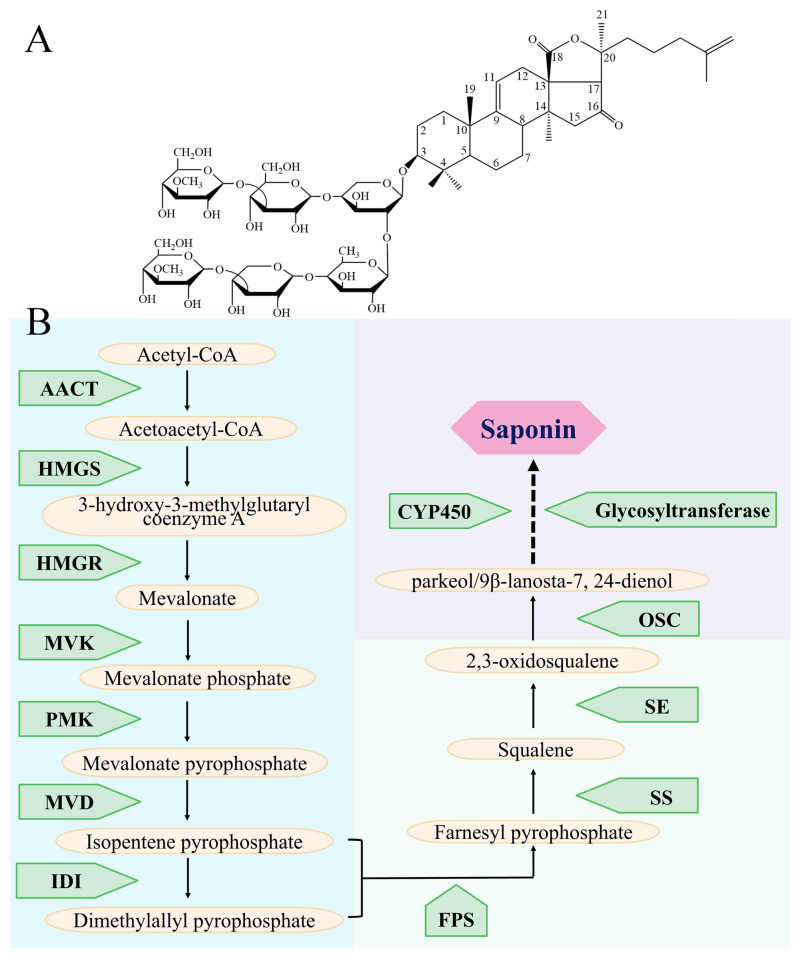
Overview of sea cucumber saponin structure and core biosynthetic pathway. (**A**) Chemical structure of Holotoxin A1, the dominant saponin in *A. japonicus*; (**B**) Core enzymatic reaction steps of the saponin biosynthetic pathway.

Figure 1 illustrates the chemical structure of Holotoxin A1, the representative saponin in *A. japonicus*, and the core enzymatic steps of the saponin biosynthetic pathway in sea cucumber to provide an intuitive framework for the following results.

On the basis of our previous studies [28,29], 13 candidate genes were screened and identified from the *A. japonicus* genome, including acetyl-CoA C-acyltransferase (AjAACT-1 and AjAACT-2), 3-hydroxy-3-methylglutaryl-CoA synthase (AjHMGS), 3-hydroxy-3-methylglutaryl-CoA reductase (AjHMGR), mevalonate kinase (AjMVK), phosphomevalonate (AjPMK) [28], pyrophosphomevalonate decarboxylase (AjMVD), isopentenyl diphosphate (AjIDI), farnesyl diphosphate synthase (AjFPS) [29], squalene synthase (AjSS), squalene monooxygenase (AjSE), and oxidosqualene cyclase (AjPS and AjLS) (our previous collaborative study [19]). From the acetylation of acetyl-CoA to the cyclization of 2,3-oxidosqualene, a molecular pathway for de novo synthesis of saponin was revealed. The full-length cDNA sequences of the candidate genes were obtained through RACE technology and submitted to GenBank (Table 1). As no signal peptides were predicted, the candidate genes were predicted to be non-secretory proteins, suggesting that they may be intracellular proteins (Figures S1 and S2, Table 1). Only AjHMGR and AjSS had transmembrane domains (Figures S1 and S2, Table 1).

### 2.2. Tissue Expression Pattern Analysis

**Figure 2:**
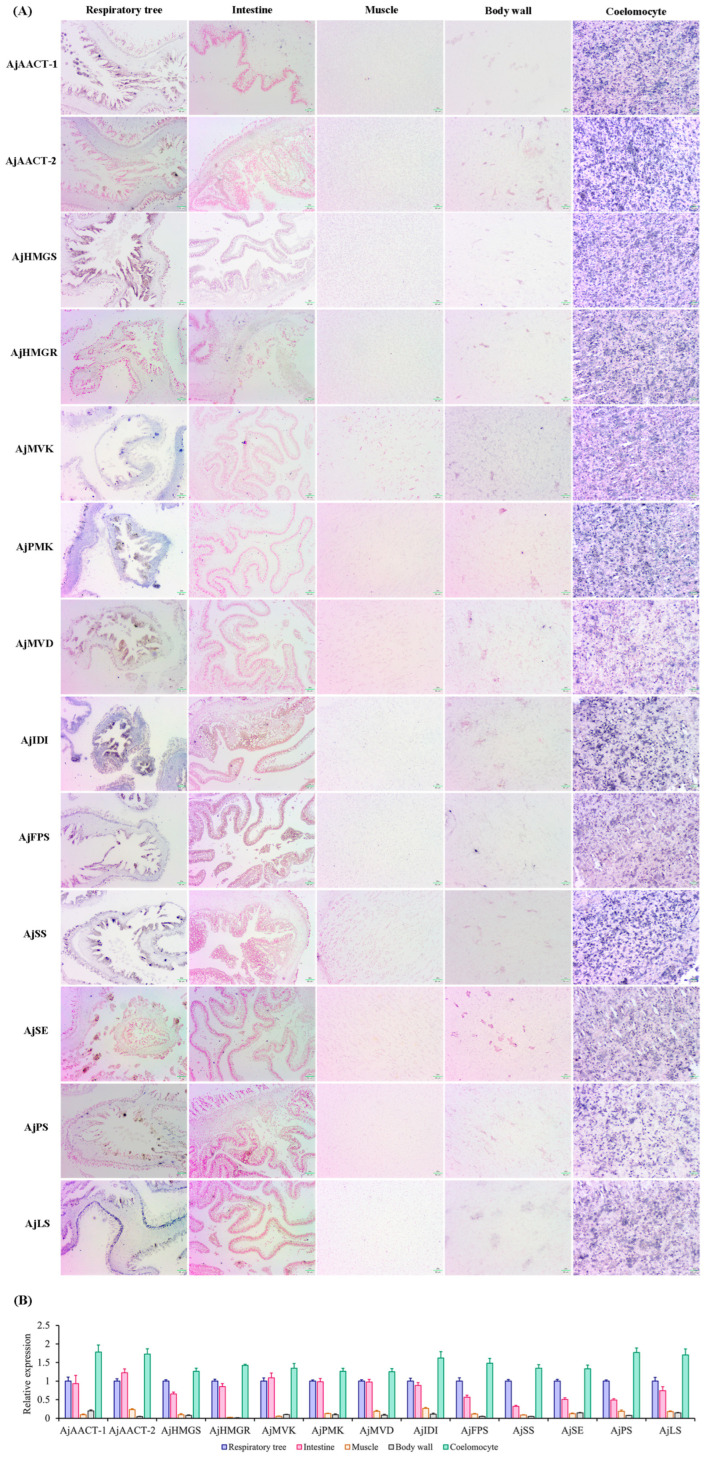
Expression of candidate genes in tissues. (**A**) Tissue distribution of candidate genes responsible for saponin biosynthesis, as measured by in situ hybridization (ISH). Scale bar = 50 μm for all panels in (**A**). (**B**) Tissue expression patterns of candidate genes responsible for saponin biosynthesis, as determined by qPCR.

On the basis of strong positive blue signals, ISH revealed high transcriptional expression levels of the 13 candidate genes in coelomocytes (Figure 2A). The ISH signals for the 13 candidate genes exhibited stronger intensity in coelomocytes than in other tissues, including respiratory tree, intestine, muscle, and body wall tissues. Moreover, no candidate gene signals were detected in control tissues. Consistent with the ISH results, qPCR analysis showed that all 13 candidate genes shared a similar tissue expression pattern, with the highest transcript abundance in coelomocytes, followed in order by respiratory tree, intestine, muscle, and body wall (Figure 2B).

### 2.3. Identification of Molecular Mechanism of Saponin Biosynthesis in A. japonicus

**Figure 3:**
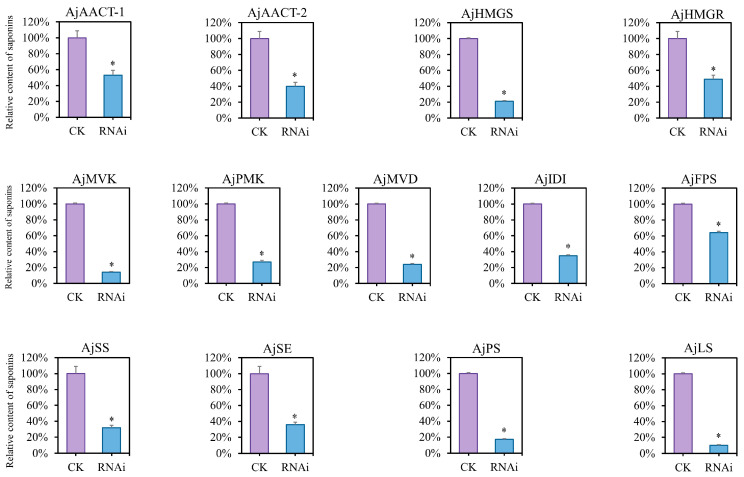
Relative saponin contents after knockdown of candidate saponin biosynthesis genes. CK, control group (sterile seawater injection), RNAi, RNA interference group (siRNA injection). Data are presented as mean ± SD. Statistical significance between CK and RNAi groups was analyzed by two-tailed Student’s *t*-test. * *p* < 0.05.

The interference rates of the siRNAs targeting the 13 candidate genes were evaluated in the coelomocyte samples (Figure S3). qPCR validation showed that all siRNAs achieved 70~80% knockdown efficiency at the mRNA level, confirming effective knockdown of each target gene. Analysis of saponin contents in the corresponding body wall samples showed that knockdown of the candidate genes exerted notable impacts on the alterations in saponin contents. It was shown that the knockdown of AjMVK, AjPS and AjLS leads to a relatively greater decrease in saponin content than the other candidate genes’ (Figure 3).

### 2.4. Exploration of the Regulatory Mechanism of Saponin Biosynthesis in A. japonicus

**Figure 4:**
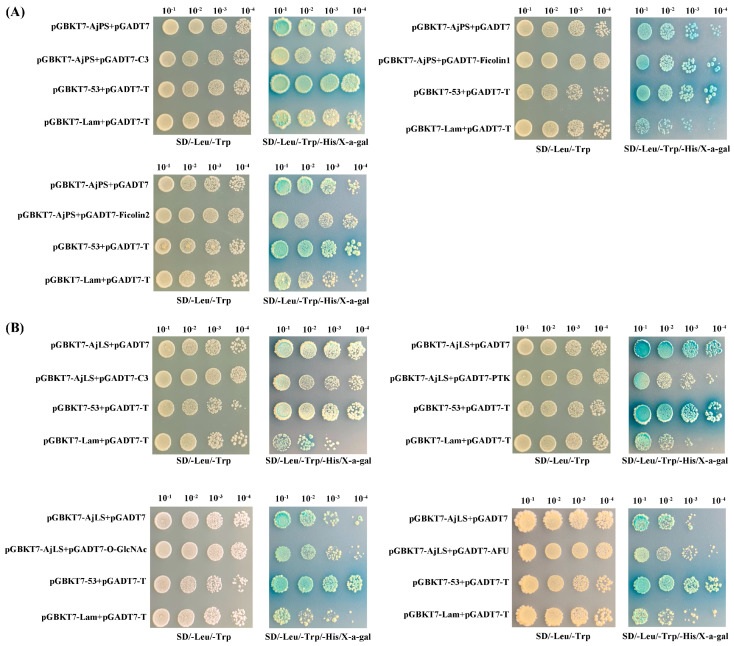
Yeast two-hybrid (Y2H) screening of AjPS (**A**) and AjLS (**B**). (**A**) Interaction of AjPS with other proteins according to Y2H screening. (**B**) Interaction of AjLS with other proteins according to Y2H screening.

The proteins interacting with AjPS and AjLS were analyzed via Yeast two-hybrid (Y2H) assays. AjPS interacted with complement component 3-2 (C3-2; PIK37564.1) and ficolins (PIK40178.1 and PIK40176.1), while AjLS interacted with C3-2, protein tyrosine kinase (PTK; PIK33117.1), O-linked beta-N-acetylglucosamine (O-GlcNAc) transferase (PIK39913.1) and α-L-fucosidase (AFU; PIK53384.1) (Figure 4A,B).

### 2.5. Verification of the Role of Regulatory Factors in the Biosynthesis of A. japonicus Saponins

**Figure 5:**
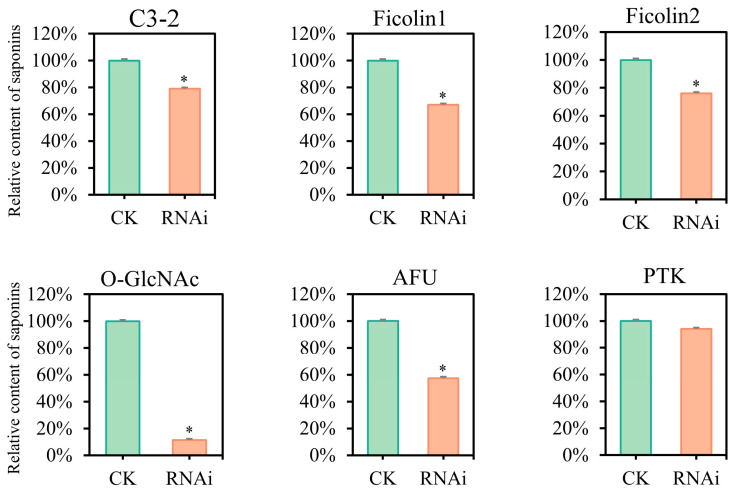
Relative saponin contents after knockdown of the identified factors. CK, control group (sterile seawater injection), RNAi, RNA interference group (siRNA injection). Data are presented as mean ± SD. Statistical significance between CK and RNAi groups was analyzed by two-tailed Student’s *t*-test. * *p* < 0.05.

**Figure 6:**
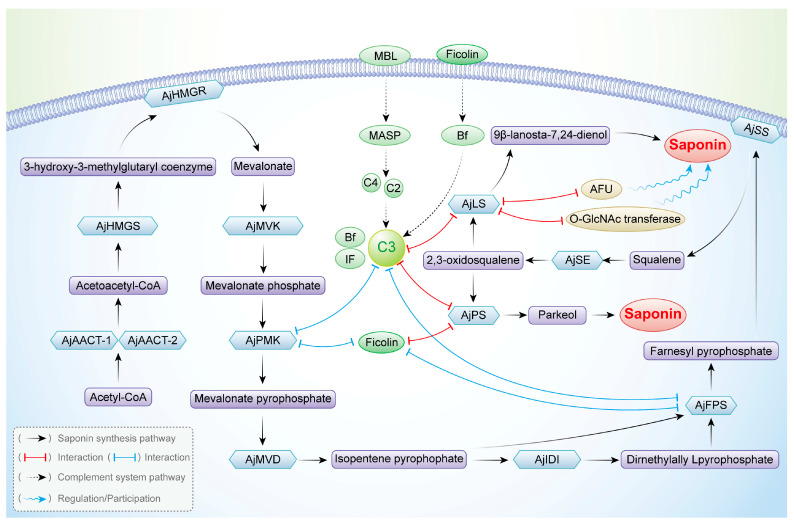
Schematic of the saponin biosynthesis pathway in *A. japonicus.* The interactions indicated by blue lines represent results from our previous studies [28,29]. The interactions indicated by red lines represent results from the present study.

The interference rates of the siRNAs targeting C3-2, Ficolin1, Ficolin2, PTK, O-GlcNAc transferase, and AFU were evaluated in the coelomocyte samples (Figure S4). qPCR validation showed that all siRNAs achieved 70~80% knockdown efficiency at the mRNA level, confirming effective knockdown of each target gene. Knockdown of C3-2, Ficolin 1, Ficolin 2, O-GlcNAc transferase, and AFU led to decreased saponin contents, suggesting that these five genes may be regulatory factors in *A. japonicus* saponin biosynthesis (Figure 5). Thus, the potential saponin biosynthetic pathway and regulatory mechanism in *A. japonicus* are assumed as depicted in Figure 6.

## 3. Discussion

At present, studies on marine saponins focus mainly on their bioactivity, chemical synthesis, and extraction process, while few studies focus on the biosynthesis pathway. *A. japonicus* is a dominant and commercially vital species in the marine aquaculture sector of China, and saponin is among the crucial traits that determine its nutritional and health-promoting value. Owing to the lack of basic research on *A. japonicus* saponins, the development and utilization of *A. japonicus* as a saponin source are limited. Thus, it is necessary to explore the de novo biosynthesis pathway of *A. japonicus* saponins to simplify the application conditions of saponins so as to promote a more extensive application of *A. japonicus* saponins in pharmaceutical, healthcare and more related industries.

In echinoderms, saponins are categorized into the *Holothuroidea* (as triterpenoid glycosides) [11,30] and *Asteroidea* (as steroidal glycosides) [31]. Both triterpenoid and steroidal saponins rely on the MVA pathway for synthesis, with 2,3-oxidosqualene serving as their common precursor. Moreover, cyclization is a vital step in the biosynthesis of triterpenoids. In general, 2,3-oxidosqualene undergoes cyclization in animals to form isomeric lanosterol, enabling cholesterol synthesis. In contrast to most animals, however, 2,3-oxidosqualene is cyclized into parkeol in certain sea cucumbers. This difference may be due to the absence of the *Cyp51* and *Dhcr7* genes in the *A. japonicus* genome, causing the loss of the ability to synthesize cholesterol [19], which aligns with the reported low cholesterol levels in sea cucumbers. Moreover, research has indicated that parkeol is transformed into glycosides in *Holothuria floridana* and *Actinopyga agassizi* through both in vivo and in vitro experimental approaches [20]. Li et al. reported that the two identified OSCs may mediate the production of parkeol (*via* AjPS) and 9β-lanosta-7,24-dienol (*via* AjLS) rather than the anticipated lanosterol [19]. Notably, parkeol has been proposed as a saponin precursor in sea cucumbers, while 9β-lanosta-7,24-dienol serves as a potential saponin precursor that requires further validation [20].

The 13 identified candidate genes are involved in the molecular pathway from acetylation of acetyl CoA to cyclization of oxidative squalene. Sequence analysis of the 13 genes revealed their intracellular localization. Additionally, ISH combined with qPCR analysis revealed that these 13 genes were highly expressed in coelomocytes. Thus, these results suggested that *A. japonicus* saponins are synthesized intracellularly and that coelomocytes are the predominant site of synthesis. Using labeled parkeol, Russell et al. confirmed that saponin biosynthesis occurs in cuvierian tubules in sea cucumbers *H. fiidecl* and *A. agassize* [20]. Cuvierian tubules, an important defensive organ possessed by some sea cucumbers of *Holothuriidae*, are located near the cloaca. This organ, specialized for saponin production, synthesizes and discharges bioactive saponins into the tubules and the body wall [32]. There are eight types of sea cucumbers in China that possess cuvierian tubules, namely *Holothuria leucospilota*, *Bohadschia argus*, *Bohadschia marmorata*, *Pearsonothuria graeffei*, *Actinopyga echinites*, *Actinopyga lecanora*, *Actinopyga mauritiana* and *Actinopyga miliaris* [33]. Because *A. japonicus* is a *Stichopodidae* species and lacks cuvierian tubules, its saponin synthesis occurs predominantly in coelomocytes, a critical immune tissue. Owing to its high saponin content, we previously used the body wall of *A. japonicus* as the extraction material for saponins [34]. However, the present findings demonstrated that the 13 saponin biosynthesis genes had low expression levels in the body wall, suggesting that there may be a transport process after saponin biosynthesis in coelomocytes. Thus, additional experiments are needed to further explore the specific mechanism. Beyond the dominant expression in coelomocytes as the primary systemic site of saponin biosynthesis, appreciable expressions of the 13 biosynthetic genes were also detected in the respiratory tree and intestine, albeit at lower levels. As mucosal interfaces in constant contact with the external aquatic environment, both respiratory tree and intestine tissues have been shown to possess immune competence in *A. japonicus*, with core immune signaling components constitutively expressed and responsive to pathogen challenge [35]. The presence of biosynthetic transcripts suggests these tissues may have the capacity for local in situ saponin synthesis, which may complement systemic production from coelomocytes and contribute to frontline mucosal defense. In the intestine specifically, gut microbiota homeostasis is tightly coupled to intestinal immune status in *A. japonicus* [36]. Notably, previous evidence has revealed a significant association between gut microbiota composition and saponin content in sea cucumbers, implying a potential interactive relationship between mucosal saponin production and gut microbial homeostasis [37]. From an ecological perspective, this local synthetic capacity may also support chemical defense against predators. Saponins are widely recognized as defensive secondary metabolites in sea cucumbers, and visceral tissues, including the intestine and respiratory tree, serve as reservoirs of these bioactive compounds [25,38]. When exposed to predation or environmental stress, sea cucumbers can expel their visceral organs via evisceration; saponins stored and synthesized locally in these tissues may be rapidly released into the surrounding environment to deter predators, representing a fast-acting defensive strategy [38]. This spatial arrangement of defensive biosynthesis aligns with the evolutionarily conserved mucosal immune strategy in marine invertebrates, where barrier tissues may autonomously produce immune effectors to cope with continuous environmental challenges [39]. The specific regulatory mechanisms and quantitative contribution of this local saponin production remain to be elucidated in further studies.

As the saponin content changed after knockdown of each candidate gene, all 13 candidate genes may be involved in the saponin biosynthesis pathway. Among these, knockdown of AjMVK, AjPS and AjLS exerted the most marked effects, suggesting that they may be the key enzymes in saponin synthesis. OSCs have been reported as key enzymes in saponin biosynthesis of different plant species, such as *Panax ginseng* and *Centella asiatica* (L.) *Urb* [40,41]. Instead of MVK, however, HMGR and SE have been identified as key enzymes of plant saponin biosynthesis [42], suggesting a difference in the underlying regulatory mechanism of saponin biosynthesis between plants and sea cucumbers. Although MVK is an important enzyme in the MVA pathway, it is located upstream of saponin biosynthesis, and the MVA pathway is also involved in the synthesis of many other substances, such as coenzyme Q10 and heme A [43]. Because OSCs in *A. japonicus* (AjPS and AjLS) are more specifically involved in saponin synthesis [19], AjPS and AjLS are better targets among the identified key enzymes to investigate the regulatory mechanism of *A. japonicus* saponin biosynthesis.

Y2H assays revealed that AjPS and AjLS interact with complement system factors, including C3-2 and ficolins. The complement system, identified across invertebrates and vertebrates, represents one of the oldest immune foundations and contributes to innate defense against common pathogens. Complement component 3 (C3) serves as a core constituent of the complement system, whereas C3-2 is an isoform of C3. C3 and C3-2 exhibit similar expression pattern, and they significantly contribute to mediating immune responses to bacterial infection in sea cucumber [44]. As members of the fibrinogen-related protein superfamily, ficolins function as pattern recognition molecules in the lectin complement pathway [45,46]. Functioning as mediators of host defense and contributing to tissue homeostasis maintenance, ficolins bind to conserved pathogen-specific structures to modify self-antigens and form complexes with pentraxins to regulate innate immune functions [46]. The interactions of key enzymes in saponin synthesis with ficolin and C3-2, implies a relationship between the lectin pathway of the complement system and saponin synthesis. Given that the biological activities of sea cucumber saponins against bacterial and fungal pathogens have been identified, it can be speculated that upon pathogen infection, *A. japonicus* may enhance saponin biosynthesis by activating the complement system to induce the cyclization process in saponin synthesis, with saponins acting as immune effector substances to kill and inhibit pathogens. Moreover, we have previously reported that AjPMK and AjFPS interact with ficolin and C3-2 [28,29], which is consistent with the conclusion that saponin biosynthesis is regulated by the lectin pathway of complement system. In addition, the skeleton structure of saponins is formed after the cyclization of 2,3-oxidosqualene. Various monomeric saponins with different structures and functions are formed via modification processes, such as hydroxylation, glycosylation, and acylation [47,48]. In the downstream stages of the saponin synthesis pathway, glycosylation of the skeleton structure is an essential process [49]. Among the identified regulatory factors of *A. japonicus* saponin synthesis, AFU and O-GlcNAc transferase are responsible for glycosylation. Moreover, AFU has been reported to play an important role in the biotransformation of saikosaponins [50]. These findings imply that AFU and O-GlcNAc transferase act as regulatory factors of *A. japonicus* saponin synthesis through interactions with AjLS and may be involved in the glycosylation of the *A. japonicus* saponin skeleton, suggesting a potential mutual regulation between upstream and downstream factors of *A. japonicus* saponin synthesis. Similarly, a potential mutual regulation has also been reported between AjPMK and AjFPS [28]. Therefore, the mutual regulation within the pathway of saponin synthesis may be an important component of the regulatory network of *A. japonicus* saponin.

Collectively, the findings presented here advance current understanding of saponin biosynthesis in *A. japonicus* relative to prior reports. Firstly, full-length cDNA validation and in vivo functional characterization were conducted for a set of 13 core genes spanning the entire saponin skeleton biosynthetic pathway, complementing earlier omics-based candidate gene predictions that have lacked direct functional confirmation. Secondly, by quantifying saponin content changes following individual gene knockdown, we delineated the relative contribution of each enzyme in the pathway and identified AjMVK, AjPS, and AjLS as key modulators of saponin accumulation. It is noteworthy that the pronounced impact of AjMVK knockdown on saponin levels differs from the well-documented paradigm in plant saponin biosynthesis, where HMGR is broadly accepted as the primary rate-limiting enzyme, pointing to lineage-specific regulatory features of the MVA pathway across plant and animal lineages. Thirdly, consistent results from ISH and qPCR analyses corroborate coelomocytes as the predominant site of saponin synthesis in *A. japonicus*, resolving ambiguity around saponin-producing tissues in sea cucumber species that do not possess Cuvierian tubules. Finally, the observed interactions between core biosynthetic enzymes and complement lectin pathway components uncover a previously uncharacterized regulatory connection between innate immunity and saponin biosynthesis, offering a molecular framework for understanding saponin-mediated chemical defense in sea cucumbers. In order to further dissect the regulatory mechanism underlying the saponin biosynthetic pathway, protein-level approaches including Western blotting and quantitative proteomics, along with in vitro activity assays for key biosynthetic enzymes, will be adopted in our follow-up research based on the transcriptional-level results obtained in the present work.

## 4. Materials and Methods

### 4.1. Experimental Sea Cucumbers

Treatment of sea cucumbers was carried out in conformity to the guidelines and regulations formulated by the Liaoning Ocean and Fisheries Science Research Institute and local government. No species under protection or endangered taxa were involved in the present study.

All experimental sea cucumbers were commercially purchased from a local aquaculture farm in Dalian, China. All individuals were identified as *A. japonicus* based on standard morphological taxonomic characteristics, including cylindrical fusiform body shape, irregularly arranged dorsal conical papillae, three longitudinal bands of tube feet on the ventral surface, and characteristic table-shaped ossicles in the body wall. Sea cucumbers were cultured in filtered seawater, and the following environmental conditions were maintained: temperature of 16 °C, salinity of 31‰, pH 8.2, and continuous aeration. Individuals (weighing 25 ± 1.76 g) were acclimated for at least one week before use.

### 4.2. RNA Preparation

Total RNA was purified from frozen samples with the RNAprep Pure Tissue Kit (TIANGEN, Beijing, China) following the manufacturer’s instructions. The quality of each RNA sample was assessed via agarose gel electrophoresis and quantitative analysis using a NanoPhotometer N50 spectrophotometer (Implen GmbH, München, Germany). The remaining RNA was stored at −80 °C for subsequent use.

### 4.3. Full-Length cDNA Cloning

Using the SMARTer RACE 5′/3′ Kit (Clontech, Mountain View, CA, USA), full-length cDNAs were cloned according to the manufacturer’s instructions, with partial sequences of candidate genes from the *A. japonicus* genome serving as the basis. Appropriate primers (Table S1) were designed using Primer 6.0. The rapid amplification of cDNA ends (RACE)-polymerase chain reaction (PCR) products was analyzed by agarose gel electrophoresis and sequenced through TA cloning (Sangon Biotech, Shanghai, China).

### 4.4. Sequence Analysis

The open reading frames of the candidate genes were analyzed with ORFfinder (https://www.ncbi.nlm.nih.gov/orffinder/; accessed on 16 June 2022). Signal peptides were predicted using the SignalP-6.0 server (https://services.healthtech.dtu.dk/services/SignalP-6.0/; accessed on 17 June 2022). The subcellular localization information was speculated by comprehensively considering the prediction results of multiple tools, including Cell-Ploc (http://www.csbio.sjtu.edu.cn/bioinf/Cell-PLoc/; accessed on 2 June 2022), and PSORT (https://www.genscript.com/psort.html; accessed on 16 June 2022). In addition, the DeepTMHMM 1.0 (https://services.healthtech.dtu.dk/services/DeepTMHMM-1.0/; accessed on 17 June 2022) was used to predict the transmembrane domain. Conserved domains were predicted using InterPro (http://www.ebi.ac.uk/interpro/; accessed on 17 June 2022).

### 4.5. Transcriptional Expression Analysis

Transcriptional expression levels of target genes were analyzed via quantitative real-time PCR (qPCR) on an Applied Biosystems 7500 Real Time PCR system (ThermoFisher Scientific, Waltham, MA, USA). First-strand cDNA was generated from 1 μg of total RNA per reaction using Evo M-MLV RT Premix for PCR (AG11706; Accurate Biotechnology (Hunan) Co., Ltd., Changsha, China), and qPCR was performed in a 20 μL reaction system using a SYBR Green Premix Pro Taq HS qPCR Kit (ROX Plus; Accurate Biotechnology (Hunan) Co., Ltd., Changsha, China). Primer 6.0 was used to design primers for target genes, and the cytochrome b (*Cytb*) gene was selected as the reference gene according to a previous report [51] (Table 2). The PCR thermocycler program was as follows: 95 °C for 30 s, 40 cycles at 95 °C for 5 s, 55 °C for 35 s and 72 °C for 25 s. Melting curve analysis was performed after cycling to confirm amplification specificity. The expression levels of target genes were all assessed in triplicates.

### 4.6. In Situ Hybridization (ISH) Assay

Transcriptional expression profiles of target genes across diverse tissues were analyzed by ISH. The probes for target genes (Table 3) were synthesized by Servicebio (China). *A. japonicus* tissues were fixed in fixative solution (Servicebio, Wuhan, China), dehydrated through a graded ethanol series, and embedded in paraffin blocks. Serial sections were cut and mounted onto glass slides and baked at 62 °C for 2 h. After deparaffinization with xylene, rehydration with anhydrous ethanol, and rinsing in diethylpyrocarbonate (DEPC)-treated water, sections were digested with proteinase K (20 μg/mL, diluted in PBS) at 37 °C for 15 min. Pre-hybridization was carried out at 37 °C for 1 h using pre-hybridization buffer (Servicebio), followed by overnight hybridization at 37 °C with digoxigenin (DIG)-labeled probes diluted to 1 μmol/L in hybridization buffer (Servicebio). Sections incubated with DIG-labeled sense probe served as negative controls. Following hybridization, slides were washed with saline sodium citrate (SSC) buffer (Servicebio), blocked with normal rabbit serum at room temperature for 30 min, and then incubated with mouse monoclonal anti-DIG antibody conjugated to alkaline phosphatase (Sigma-Aldrich, St. Louis, MO, USA) at 37 °C for 40 min. After rinsing with Tris-buffered saline (TBS), chromogenic signals were developed using 5-bromo-4-chloro-3-indolyl phosphate (BCIP)/nitro blue tetrazolium (NBT) solution, and the reaction was terminated by rinsing with distilled water. Nuclei were counterstained with 0.1% nuclear fast red solution. Finally, sections were air-dried, mounted with neutral balsam, and observed under a bright-field microscope (Nikon Eclipse Ci, Tokyo, Japan). Positive hybridization signals appeared as blue/purple staining, with color intensity corresponding to relative transcript abundance. The assay was performed with minor modifications based on a previously established protocol [52].

### 4.7. RNA Interference

Small interfering RNAs (siRNAs) were designed and synthesized for each target gene by Genepharma (Shanghai, China) based on the obtained full-length cDNA sequences (Table 2). Each sea cucumber in the experimental group received 100 μL of 25 μM siRNA solution via coelomic cavity injection, and control individuals were injected with an equal volume of sterile seawater through the same route. Fifteen individuals per group were sampled at 24, 48, 72, 96, 120, 144, and 168 h after the first injection. A second booster injection was administered immediately after sampling at 72 h to counteract siRNA degradation in vivo and maintain stable knockdown efficiency over the full time course. At each time point, body walls and coelomocytes from sampled individuals were pooled into mixed samples. Coelomocyte samples were used to evaluate siRNA-mediated target gene knockdown efficiency via qPCR, with all measurements performed in triplicate.

### 4.8. Detection of Saponin Contents

Only when the siRNA efficiency fell within the range of 70% to 80% were the saponin contents in the body wall samples evaluated using microwave-assisted extraction, followed by high-performance liquid chromatography (HPLC)-diode-array detection. The specific experimental parameters and operational details are the same as those reported in our previous study [34]. Samples were measured in triplicate.

### 4.9. Yeast Two-Hybrid Library Screening

Y2H screening was performed using the Matchmaker Two Hybrid system (Clontech, Mountain View, CA, USA). A cDNA library was constructed with CloneMiner II cDNA library construction kit (Invitrogen, Carlsbad, CA, USA) by cloning the full-length cDNA library from mRNAs of the *A. japonicus* coelomocytes. The coding sequences CDSs of the key saponin biosynthesis genes were cloned into the pGBKT7 bait vector. Y2HGold competent yeast cells with pGBKT7-AjPS/AjLS were used to screen the library after the auto-activation test. For the positive control, pGBKT7-53 and pGADT7 were co-transformed, whereas pGBKT7-Lam combined with pGADT7 served as the negative control. DDO/X (SD/-Trp/-Leu/X-α-Gal) and TDO/X (SD/-Trp/-Leu/-His/X-α-Gal) agar plates were used to screen the transformants. Individual blue colonies were isolated and inoculated onto higher-stringency QDO/X/A plates (SD/-Trp/-Leu/-His/-Ade/X-α-Gal/AbA) to test reporter gene expression. From putatively positive clones, prey plasmids were extracted and sequenced. To confirm interactions, pGBKT7-AjPS/AjLS was co-transformed with plasmids from presumed positive prey into Y2HGold cells. In brief, prey plasmids were isolated from putatively positive clones, and each plasmid was introduced into *E. coli* DH5α competent cells. The plasmids were then purified from transformants growing on selective Luria Broth (LB)/ampicillin agar plates. Each putatively positive prey plasmid was co-introduced with pGBKT7-AjPS/AjLS and pGBKT7 into Y2HGold cells, and co-transformants were grown on DDO/X plates to assess interactions. For controls, co-transformants containing pGADT7-T and pGBKT7-Lam served as the negative control on QDO/X/A plates, while the positive control comprised co-transformants with pGADT7-T and pGBKT7-53 on the same selective medium. True positive interactions were indicated by the appearance of blue colonies. Positive prey plasmids were sequenced, and the resulting sequences were subjected to BLAST analysis using National Center for Biotechnology Information databases (https://blast.ncbi.nlm.nih.gov/Blast.cgi; accessed on 22 June 2022).

### 4.10. Statistical Analysis

The Relative Expression Software Tool 384 v.2 [53] was employed to analyze the transcriptional expression levels of target genes in different tissues with respect to respiratory tree tissue as well as the relative mRNA abundances following RNAi. A two-tailed Student’s *t*-test was used for comparisons between two independent groups. A *p*-value less than 0.05 was considered statistically significant. All statistical analyses were performed using GraphPad Prism 9 software (GraphPad Software, San Diego, CA, USA).

## 5. Conclusions

The present study elucidated the molecular pathway underlying the de novo synthesis of the saponin skeleton in *A. japonicus*—from the acetylation of acetyl-CoA to the cyclization of 2,3-oxidosqualene. Saponins are predominantly synthesized in *A. japonicus* coelomocytes. Moreover, AjMVK, AjPS, and AjLS are key enzymes involved in saponin synthesis. Further, *A. japonicus* saponin biosynthesis may be regulated by the lectin pathway of the complement system and may be affected by glycosylation enzymes, including O-GlcNAc transferase and AFU.

## Figures and Tables

**Table 1:** Sequence analysis information for candidate saponin biosynthesis genes.

Proteins	GenBank Numbers	Amino Acids	Subcellular Localization	Signal Peptide Protein	Transmembrane Helices
AjAACT-1	MZ923505	394 aa	intracellular	no	no
AjAACT-2	MZ923506	417 aa	intracellular	no	no
AjHMGS	OL440162	516 aa	intracellular	no	no
AjHMGR	MZ923507	899 aa	intracellular	no	yes
AjMVK	MW930707	341 aa	intracellular	no	no
AjPMK	MW930708 [28]	203 aa	intracellular	no	no
AjMVD	MW930709	398 aa	intracellular	no	no
AjIDI	MW930710	276 aa	intracellular	no	no
AjFPS	MW930711 [29]	434 aa	intracellular	no	no
AjSS	MW930705	403 aa	intracellular	no	yes
AjSE	MW930706	513 aa	intracellular	no	no
AjPS	PIK43102.1 [19]	657 aa	intracellular	no	no
AjLS	PIK43101.1 [19]	568 aa	intracellular	no	no

**Table 2:** Sequences of primers used in the present study.

Primer Name	Sequence (5′–3′)	Sequence Source
AjAACT-1-RT-F	CGGGAACATCATCAGGTATC	Designed in this study
AjAACT-1-RT-R	CTGTGCCCAAGCAACTAC	Designed in this study
AjAACT-2-RT-F	AGGTTGAATGTGGAGCCGTT	Designed in this study
AjAACT-2-RT-R	ACCACACTGAAAGCCTCGTT	Designed in this study
AjHMGS-RT-F	GATGGAGAGAGGTTTACGAGG	Designed in this study
AjHMGS-RT-R	CCCAAGGTAGCACTGGATG	Designed in this study
AjHMGR-RT-F	CAATGCCCATGCTGCGAAC	Designed in this study
AjHMGR-RT-R	TCGATTGAGGGCATCGTA	Designed in this study
AjMVK-RT-F	GCAGGTCTCAGCGACGATATAACA	Designed in this study
AjMVK-RT-R	CGGCAGGTTAGATGTAGCGAGAG	Designed in this study
AjPMK-RT-F	CCACAGAGTACAAGGAGCAGCACAG	Designed previously [28]
AjPMK-RT-R	TTCGCGCATCACTGATGATCCAGAC	Designed previously [28]
AjMVD-RT-F	AGAGCAACCAACTCCACTCTGTCTG	Designed in this study
AjMVD-RT-R	ATTGTAGGCATGTACGAGCCTCAC	Designed in this study
AjIDI-RT-F	GGCAGCCCAGAGGAAATTGAAACAT	Designed in this study
AjIDI-RT-R	TGTCGCAAGGTGCCTGGTAGT	Designed in this study
AjFPS-RT-F	TCTACGTGGAAGCAGCCATCTACA	Designed previously [29]
AjFPS-RT-R	TGTCTCGGAGGTCATCAGGTCTAAC	Designed previously [29]
AjSS-RT-F	TCATGATGGAAGCCACGGAC	Designed in this study
AjSS-RT-R	AAGTTTGGGTCGTCTCTCGG	Designed in this study
AjSE-RT-F	CCCTACCCATCACAGGAGGA	Designed in this study
AjSE-RT-R	GTGACCACGCCTTCGATGTA	Designed in this study
AjPS-RT-F	ATGCTGATTCGGTGGCTTGA	Designed in this study
AjPS-RT-R	CGGCCCATCCAGATGTAGTC	Designed in this study
AjLS-RT-F	TGGCCATCCCAGAGAGACAA	Designed in this study
AjLS-RT-R	GGGTCAACCTAAACGGCTCA	Designed in this study
C3-2-RT-F	TGCCCTCCTTGTTTTGATAG	Designed in this study
C3-2-RT-R	GTTGCGTTGACGACTTTGTA	Designed in this study
Ficolin1-RT-F	CAACGGGAACTGTCACTCA	Designed in this study
Ficolin1-RT-R	GGCGAATTTTCATCTCAAC	Designed in this study
Ficolin2-RT-F	ATCCCAACGGACAATCTG	Designed in this study
Ficolin2-RT-R	AAACACCCGCATCCACTC	Designed in this study
O-GlcNAc-RT-F	AGCCAGAATGAAGCGACT	Designed in this study
O-GlcNAc-RT-R	ACGATGGGCTATGGATTT	Designed in this study
AFU-RT-F	TGTTTACTCTGTCCCTGCTT	Designed in this study
AFU-RT-R	GCGGATAGTTTTCCTTCATA	Designed in this study
PTK-RT-F	AGGTTACAGGATGCCTCGTC	Designed in this study
PTK-RT-R	TGTCTTCGGGATTTTCACTC	Designed in this study
Cytb-RT-F	TGAGCCGCAACAGTAATC	[51]
Cytb-RT-R	AAGGGAAAAGGAAGTGAAAG	[51]
rAjMVK-F	TTTTGGATCCATGTTCAGTGCACTGGTG	Designed in this study
rAjMVK-R	TCTCCTCGAGTTAGGCGGTGAAATTATTCG	Designed in this study
AjAACT-1-siRNA-sense	GCUGAAAGAUACUCUCAUUTT	Designed in this study
AjAACT-1-siRNA-antisense	AAUGAGAGUAUCUUUCAGCTT	Designed in this study
AjAACT-2-siRNA-sense	GCGGCCAUCAGGAUGUAAUTT	Designed in this study
AjAACT-2-siRNA-antisense	AUUACAUCCUGAUGGCCGCTT	Designed in this study
AjHMGS-siRNA-sense	GGAAUCGAGUUCCUGGGAUTT	Designed in this study
AjHMGS-siRNA-antisense	AUCCCAGGAACUCGAUUCCTT	Designed in this study
AjHMGR-siRNA-sense	GCGAACAUAGUCACAGCAATT	Designed in this study
AjHMGR-siRNA-antisense	UUGCUGUGACUAUGUUCGCTT	Designed in this study
AjMVK-siRNA-sense	GGUACCAUUCCUGUACCAUTT	Designed in this study
AjMVK-siRNA-antisense	AUGGUACAGGAAUGGUACCTT	Designed in this study
AjPMK-siRNA-sense	GCGAAGAAAGAGUGACAUUTT	Designed in this study
AjPMK-siRNA-antisense	AAUGUCACUCUUUCUUCGCTT	Designed in this study
AjMVD-siRNA-sense	GCAACAGAAUGCUGUGAAATT	Designed in this study
AjMVD-siRNA-antisense	UUUCACAGCAUUCUGUUGCTT	Designed in this study
AjIDI-siRNA-sense	GGUACCCUUAGAAGACUUUTT	Designed in this study
AjIDI-siRNA-antisense	AAAGUCUUCUAAGGGUACCTT	Designed in this study
AjFPS-siRNA-sense	GCUGACGGAUGCAAAUUUATT	Designed previously [29]
AjFPS-siRNA-antisense	UAAAUUUGCAUCCGUCAGCTT	Designed previously [29]
AjSS-siRNA-sense	GCACGGGAUGAGCGAAUUUTT	Designed in this study
AjSS-siRNA-antisense	AAAUUCGCUCAUCCCGUGCTT	Designed in this study
AjSE-siRNA-sense	CCCAGUUUCUCCUCCCGUUTT	Designed in this study
AjSE-siRNA-antisense	AACGGGAGGAGAAACUGGGTT	Designed in this study
AjPS-siRNA-sense	GCAACCCUGUCAUUCCCUATT	Designed in this study
AjPS-siRNA-antisense	UAGGGAAUGACAGGGUUGCTT	Designed in this study
AjLS-siRNA-sense	GCCCAGAAGAUGGAGACAUTT	Designed in this study
AjLS-siRNA-antisense	AUGUCUCCAUCUUCUGGGCTT	Designed in this study
C3-2-siRNA-sense	CGCCAUGCUAUAAAUCAAUTT	Designed in this study
C3-2-siRNA-antisense	AUUGAUUUAUAGCAUGGCGTT	Designed in this study
Ficolin1-siRNA-sense	GACUGAUCUGUGAAGACUATT	Designed in this study
Ficolin1-siRNA-antisense	UAGUCUUCACAGAUCAGUCTT	Designed in this study
Ficolin2-siRNA-sense	GGAGUACGAAAGUGUUAUUTT	Designed in this study
Ficolin2-siRNA-antisense	AAUAACACUUUCGUACUCCTT	Designed in this study
O-GlcNAc-siRNA-sense	GCGUUCACAUCCUAAUCAATT	Designed in this study
O-GlcNAc-siRNA-antisense	UUGAUUAGGAUGUGAACGCTT	Designed in this study
AFU-siRNA-sense	CACGGUUCGUAGAGUUUAUTT	Designed in this study
AFU-siRNA-antisense	AUAAACUCUACGAACCGUGTT	Designed in this study
PTK-siRNA-sense	GUGAGGGUGUCCGUAUAUATT	Designed in this study
PTK-siRNA-antisense	UAUAUACGGACACCCUCACTT	Designed in this study

**Table 3:** Sequences of probes used in the present study.

Probe Name	Sequence
AjAACT-1	5′-DIG-GCUGUCCCCAAACCUGAACUCACUCCUC-3′
AjAACT-2	5′-DIG-UUCAAUGUCUGGUUUACCUCUCUUCUGU-3′
AjHMGS	5′-DIG-GAGCCCCAAGGUAGCACUGGAUGGAAAG-3′
AjHMGR	5′-DIG-CUCUGUACCCUUAGAGACCAUGUUCAUC-3′
AjMVK	5′-DIG-AUACAUCCGUCCAUUUUUCCUACCACAA-3′
AjPMK	5′-DIG-CAUCAUCGAUACCUUUCGUGGGCACUA-3′
AjMVD	5′-DIG-CGUCUCGUUUUCCCCAAUAUUUUACUACA-3′
AjIDI	5′-DIG-GGUGCCUGGUAGUGGACCCCUGGUUAUG-3′
AjFPS	5′-DIG-CACCUGGUCCACGCCGUAGUUAUCCUUUA-3′
AjSS	5′-DIG-GCUCAUCCCGUGCCCCAUUCUCUUUG-3′
AjSE	5′-DIG-AUUGCUUCAUAGUCUCCUACAUUCUUCAUU-3′
AjPS	5′-DIG-CGACGAAUAAUUCCUUUCUCAGGCUUAAGA-3′
AjLS	5′-DIG-CAUCUUGGCGGUAGUCAGGAUCAGAAUC-3′

## Data Availability

The data supporting the findings of this study are included in the article and Supplementary Materials. Further inquiries can be directed to the corresponding authors.

## Supplementary Materials

The following supporting information can be downloaded at: https://www.mdpi.com/article/10.3390/md24070230/s1, Figure S1: Signal peptides predictions of candidate genes responsible for saponin biosynthesis through the SignalP-6.0 server; Figure S2: Transmembrane domain predictions of candidate genes responsible for saponin biosynthesis through the DeepTMHMM-1.0; Figure S3: Relative expression levels after knockdowns of candidate genes responsible for saponin biosynthesis; * *p* < 0.05, significantly different as compared with the control group (CK, sterile seawater injection); RNAi, the RNA interference group (siRNA injection); Figure S4: Relative expression levels after knockdowns of the identified factors; * *p* < 0.05, significantly different as compared with the control group (CK, sterile seawater injection); RNAi, the RNA interference group (siRNA injection); Table S1: The primer sequences for RACE used in this study.

## References

[1] Yu Z., Zhou Y., Yang H., Hu C. (2014). Bottom culture of the sea cucumber *Apostichopus japonicus* Selenka (Echinodermata: Holothuroidea) in a fish farm, southern China. Aquac. Res..

[2] Fu X., Xue C., Miao B., Li Z., Gao X., Yang W. (2005). Characterization of proteases from the digestive tract of sea cucumber (*Stichopus japonicus*): High alkaline protease activity. Aquaculture.

[3] Malaiwong N., Chalorak P., Jattujan P., Manohong P., Niamnont N., Suphamungmee W., Sobhon P., Meemon K. (2019). Anti-Parkinson activity of bioactive substances extracted from *Holothuria leucospilota*. Biomed. Pharmacother..

[4] Zhao Y., Xue C., Zhang T., Wang Y. (2018). Saponins from sea cucumber and their biological activities. J. Agric. Food Chem..

[5] Zhang X., Han L., Sheng W., Li X., Zhang S., Xia Q., Yang G.E., Liu K. (2019). Two novel holostane-type glycosides from the viscera of sea cucumber *Apostichopus japonicus* with antitumor activities. Rev. Roum. Chim..

[6] Abul H., Deepika D., Fereidoon S. (2020). Northern Sea Cucumber (*Cucumaria frondosa*): A Potential Candidate for Functional Food, Nutraceutical, and Pharmaceutical Sector. Mar. Drugs.

[7] Khattab R.A., Elbandy M., Lawrence A., Paget T., Rae-Rho J., Binnaser Y.S., Ali I. (2018). Extraction, identification and biological activities of saponins in sea cucumber *Pearsonothuria graeffei*. Comb. Chem. High Throughput Screen..

[8] Stonik V.A. (2001). Marine polar steroids. Russ. Chem. Rev..

[9] Francis G., Kerem Z., Makkar H.P.S., Becker K. (2002). The biological action of saponins in animal systems: A review. Br. J. Nutr..

[10] Danielle S. (2008). Deep-sea natural products. Nat. Prod. Rep..

[11] Mitu S., Bose U., Suwansa-ard S., Turner L., Zhao M. (2017). Evidence for a Saponin Biosynthesis Pathway in the Body Wall of the Commercially Significant Sea Cucumber *Holothuria scabra*. Mar. Drugs.

[12] Chappell J. (2002). The genetics and molecular genetics of terpene and sterol origami. Curr. Opin. Plant Biol..

[13] Thimmappa R., Geisler K., Louveau T., O’Maille P., Osbourn A. (2014). Triterpene Biosynthesis in Plants. Annu. Rev. Plant Biol..

[14] Masaaki S., Yuji K., Miyuki O., Zhang H., Yutaka E. (2008). Identification of a product specific amyrin synthase from *Arabidopsis thaliana*. Plant Physiol. Biochem..

[15] Xue Z., Tan Z., Huang A., Zhou Y., Sun J., Wang X., Thimmappa R.B., Stephenson M.J., Osbourn A., Qi X. (2018). Identification of key amino acid residues determining product specificity of 2,3-oxidosqualene cyclase in *Oryza* species. New Phytol..

[16] Yang J., Hu Z., Zhang T., Gu A., Gong T., Zhu P. (2018). Progress on the studies of the key enzymes of ginsenoside biosynthesis. Molecules.

[17] Liu H., Kong X., Chen J., Zhang H. (2018). De novo sequencing and transcriptome analysis of *Stichopus horrens* to reveal genes related to biosynthesis of triterpenoids. Aquaculture.

[18] Yang Y., Li X., Sun L. (2021). Triterpenoid saponin biosynthesis genes and their expression patterns during the development of sea cucumber *Apostichopus japonicus*. J. Oceanol. Limnol..

[19] Li Y., Wang R., Xun X., Wang J. (2018). Sea cucumber genome provides insights into saponin biosynthesis and aestivation regulation. Cell Discov..

[20] Kerr R.G., Chen Z. (1995). In vivo and in vitro biosynthesis of saponins in sea cucumbers. J. Nat. Prod..

[21] Zaynab M., Sharif Y., Abbas S., Afzal M.Z., Qasim M., Khalofah A., Ansari M.J., Khan K.A., Tao L., Li S. (2021). Saponin toxicity as key player in plant defense against pathogens. Toxicon.

[22] Gruiz K., Waller G.R., Yamasaki K. (1996). Fungitoxic activity of saponins: Practical use and fundamental principles. Saponins Used in Traditional and Modern Medicine.

[23] Nawrot J., Harmatha J. (2012). Phytochemical feeding deterrents for stored product insect pests. Phytochem. Rev..

[24] Thimmappa R., Wang S., Zheng M., Misra R.C., Huang A.C., Saalbach G., Chang Y., Zhou Z., Hinman V., Bao Z. (2022). Biosynthesis of saponin defensive compounds in sea cucumbers. Nat. Chem. Biol..

[25] Dyck S.V., Caulier G., Todesco M., Gerbaux P., Fournier I., Wisztorski M., Flammang P. (2011). The triterpene glycosides of *Holothuria forskali:* Usefulness and efficiency as a chemical defense mechanism against predatory fish. J. Exp. Biol..

[26] Fagbohun O.F., Joseph J.S., Oriyomi O.V., Rupasinghe H.P.V. (2023). Saponins of North Atlantic Sea Cucumber: Chemistry, Health Benefits, and Future Prospectives. Mar. Drugs.

[27] Dufayet L., Caré W., Haro L., Ameltchenko M., Knezynski M., Vodovar D., Langrand J. (2021). Acute occupational exposure to holothurians (*Cucumaria frondosa*) resulting in irritating symptoms: About three cases. Toxicon.

[28] Jiang P., Gao S., Chen Z., Sun H., Li P., Yue D., Pan Y., Wang X., Mi R., Dong Y. (2022). Cloning and characterization of a phosphomevalonate kinase gene that is involved in saponin biosynthesis in the sea cucumber *Apostichopus japonicus*. Fish Shellfish Immunol..

[29] Jiang P., Chen Z., Gao S., Zhang F., Li L., Liu Y., Li P., Xiao Y., Dong Y., Liu G. (2024). A farnesyl pyrophosphate synthase from *Apostichopus japonicus* is involved in saponin biosynthesis and regulated by immune processes. Aquac. Res..

[30] Dyck S.V., Pascal G., Patrick F. (2009). Elucidation of molecular diversity and body distribution of saponins in the sea cucumber *Holothuria forskali* (Echinodermata) by mass spectrometry. Comp. Biochem. Physiol. Part. B Biochem. Mol. Biol..

[31] Marie D., Maxence W., Corentin D., De W.J., Guillaume C., Elise H., Igor E., Isabelle F., Patrick F., Pascal G. (2015). Inter- and intra-organ spatial distributions of sea star saponins by MALDI imaging. Anal. Bioanal. Chem..

[32] Kawita C., Pawanrat C., Worawit S., Prasert S., Krai M. (2019). Saponin-enriched extracts from body wall and cuvierian tubule of *Holothuria leucospilota* reduce fat accumulation and suppress lipogenesis in Caenorhabditis elegans: Anti-obesity effects of *H. leucospilota*-derived saponins. J. Sci. Food Agric..

[33] Chen T., Ren C., Wong N.-K., Yan A., Sun C., Fan D., Luo P., Jiang X., Zhang L., Ruan Y. (2023). The *Holothuria leucospilotagenome* elucidates sacrificial organ expulsion and bioadhesive trap enriched with amyloid-patterned proteins. Proc. Natl. Acad. Sci. USA.

[34] Guiying L., Xuda W., Kun G., Jinhao W., Zhaohui W., Jing D., Lun S., Zunchun Z. (2022). Isolation, Identification, and Quantitative Determination of Saponin in *Apostichopus japonicus* by HPLC-DAD. J. Ocean Univ. China.

[35] Shao Y., Li C., Zhang W., Duan X., Li Y., Han Q., Jin C. (2015). Three members in JAK/STAT signal pathway from the sea cucumber *Apostichopus japonicus*: Molecular cloning, characterization and function analysis. Fish Shellfish Immunol..

[36] Cui L., Xie Y., Luo K., Wang M., Liu L., Li C., Tian X. (2025). Physiological and intestinal microbiota responses of sea cucumber *Apostichopus japonicus* to various stress and signatures of intestinal microbiota dysbiosis. Front. Microbiol..

[37] Zhao Z., Jiang J., Zheng J., Pan Y., Dong Y., Chen Z., Gao S., Xiao Y., Jiang P., Wang X. (2022). Exploiting the gut microbiota to predict the origins and quality traits of cultured sea cucumbers. Environ. Microbiol..

[38] Liu Y., Lu Z., Yan Z., Lin A., Han S., Li Y., Yang X., Li X., Yin X., Zhang R. (2023). Sea Cucumber Viscera Contains Novel Non-Holostane-Type Glycoside Toxins that Possess a Putative Chemical Defense Function. J. Chem. Ecol..

[39] Saco A., Rey-Campos M., Rosani U., Novoa B., Figueras A. (2021). The Evolution and Diversity of Interleukin-17 Highlight an Expansion in Marine Invertebrates and Its Conserved Role in Mucosal Immunity. Front. Immunol..

[40] Han J.Y., Kwon Y.S., Yang D.C., Jung Y.R., Choi Y.E. (2007). Expression and RNA Interference-Induced Silencing of the Dammarenediol Synthase Gene in *Panax ginseng*. Plant Cell Physiol..

[41] Kim O., Kim M., Hwang S., Ahn J., Hwang B. (2005). Cloning and molecular analysis of cDNA encoding cycloartenol synthase from *Centella asiatica* (L.) urban. Biotechnol. Bioprocess. Eng..

[42] Hou M., Wang R., Zhao S., Wang Z. (2021). Ginsenosides in Panax genus and their biosynthesis. Acta Pharm. Sin. B.

[43] Buhaescu I., Izzedine H. (2007). Mevalonate pathway: A review of clinical and therapeutical implications. Clin. Biochem..

[44] Zhou Z., Sun D., Yang A., Dong Y., Chen Z., Wang X., Guan X., Jiang B., Wang B. (2011). Molecular characterization and expression analysis of a complement component 3 in the sea cucumber (*Apostichopus japonicus*). Fish Shellfish Immunol..

[45] Liu Y., Zhang A., Guo N., Hao Q., Li F. (2022). A pattern recognition receptor ficolin from *Portunus trituberculatus* (Ptficolin) regulating immune defense and hemolymph coagulation. Int. J. Biol. Macromol..

[46] Garred P., Genster N., Genster N., Bayarri-Olmos R., Rosbjerg A., Ma Y.J., Skjoedt M.-O. (2016). A journey through the lectin pathway of complement—MBL and beyond. Immunol. Rev..

[47] Haralampidis K., Trojanowska M., Osbourn A.E. (2002). Biosynthesis of triterpenoid saponins in plants. Adv. Biochem. Eng. Biotechnol..

[48] Bowles D., Isayenkova J., Lim E.-K., Poppenberger B. (2005). Glycosyltransferases: Managers of small molecules. Curr. Opin. Plant Biol..

[49] Chen Y., Yan Q., Ji Y., Bai X., Li D., Mu R., Guo K., Yang M., Tao Y., Gershenzon J. (2023). Unraveling the serial glycosylation in the biosynthesis of steroidal saponins in the medicinal plant *Paris polyphylla* and their antifungal action. Acta Pharm. Sin. B.

[50] Hiroaki K., Teruaki A., Meselhy M., Masao H. (1997). Enzymes responsible for the metabolism of saikosaponins from *Eubacterium* sp. A-44, a human intestinal anaerobe. Biol. Pharm. Bull..

[51] Dong Y., Sun H., Zhou Z., Yang A., Chen Z., Guan X., Gao S., Wang B., Jiang B., Jiang J. (2014). Expression Analysis of Immune Related Genes Identified from the Coelomocytes of Sea Cucumber (*Apostichopus japonicus*) in Response to LPS Challenge. Int. J. Mol. Sci..

[52] Jiang J., Gao S., Wang X., Guan X., Wang B., Chen Z., Zhao Z., Sun H., Dong Y., Zhou Z. (2022). The role of a novel secretory peptidoglycan recognition protein from the sea cucumber *Apostichopus japonicus* in innate immunity. Aquaculture.

[53] Pfaffl M.W., Horgan G.W., Dempfle L. (2002). Relative expression software tool (REST©) for group-wise comparison and statistical analysis of relative expression results in real-time PCR. Nucleic Acids Res..

