# Supplementary material for: Molecular Pathway and Regulatory Mechanism of the Saponin Biosynthesis in Sea Cucumber Apostichopus japonicus
Source: Mar Drugs. 2026 Jun 30;24(7):230. doi: 10.3390/md24070230 (PMC13412492; doi:10.3390/md24070230)
Supplement: Supplementary file 1 [file marinedrugs-24-00230-s001.zip › marinedrugs-4375929-supplementary.pdf]

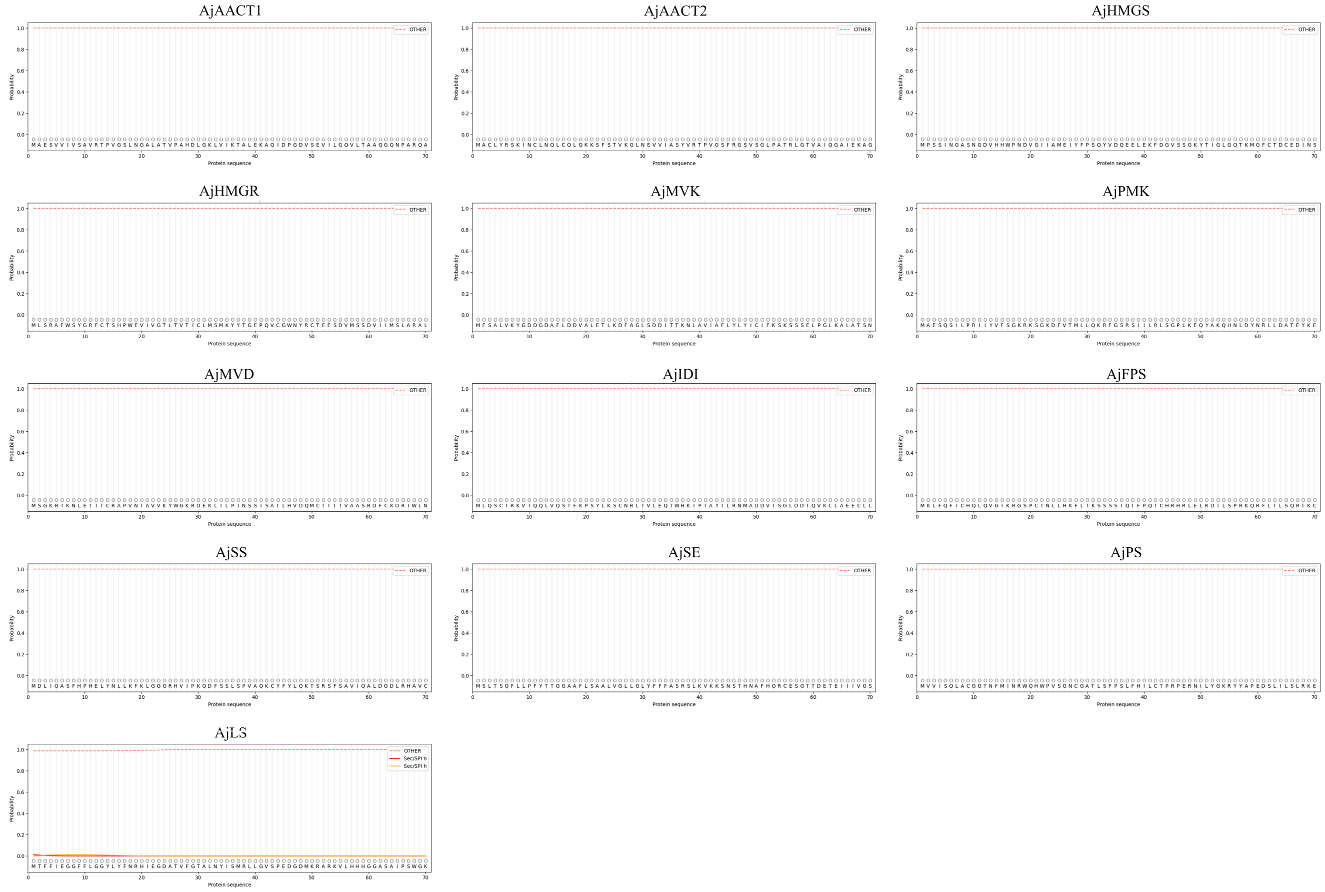

Figure S1. Signal peptides predictions of candidate genes responsible for saponin biosynthesis through the SignalP-6.0 server.

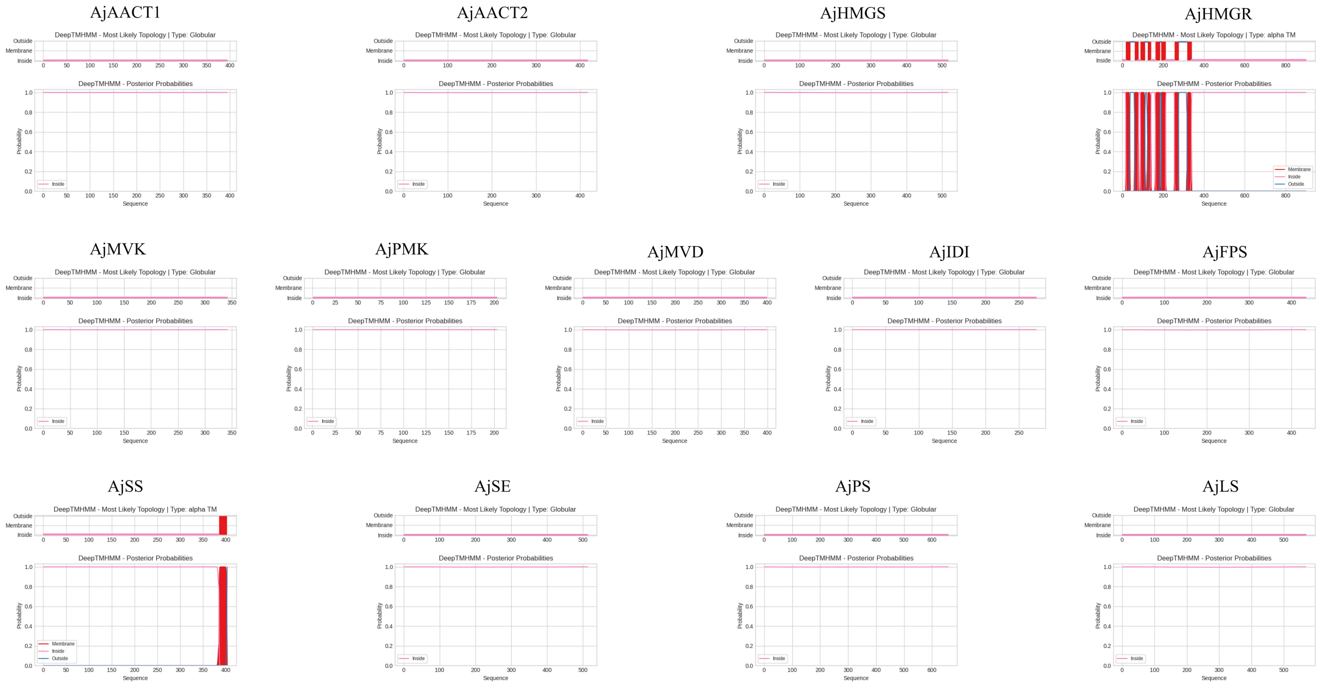

Figure S2. Transmembrane domain predictions of candidate genes responsible for saponin biosynthesis through the DeepTMHMM-1.0.

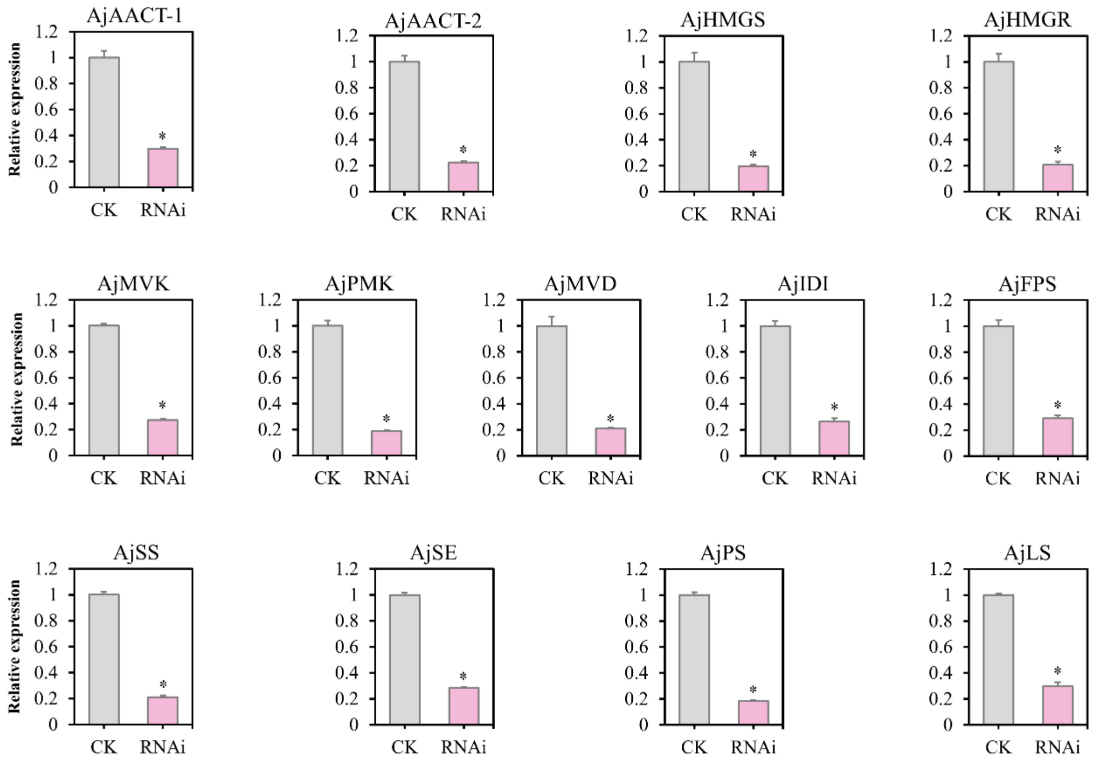

Figure S3. Relative expression levels after knockdowns of candidate genes responsible for saponin biosynthesis; $*p<0.05$, significantly different as compared with the control group (CK, sterile seawater injection); RNAi, the RNA interference group (siRNA injection).

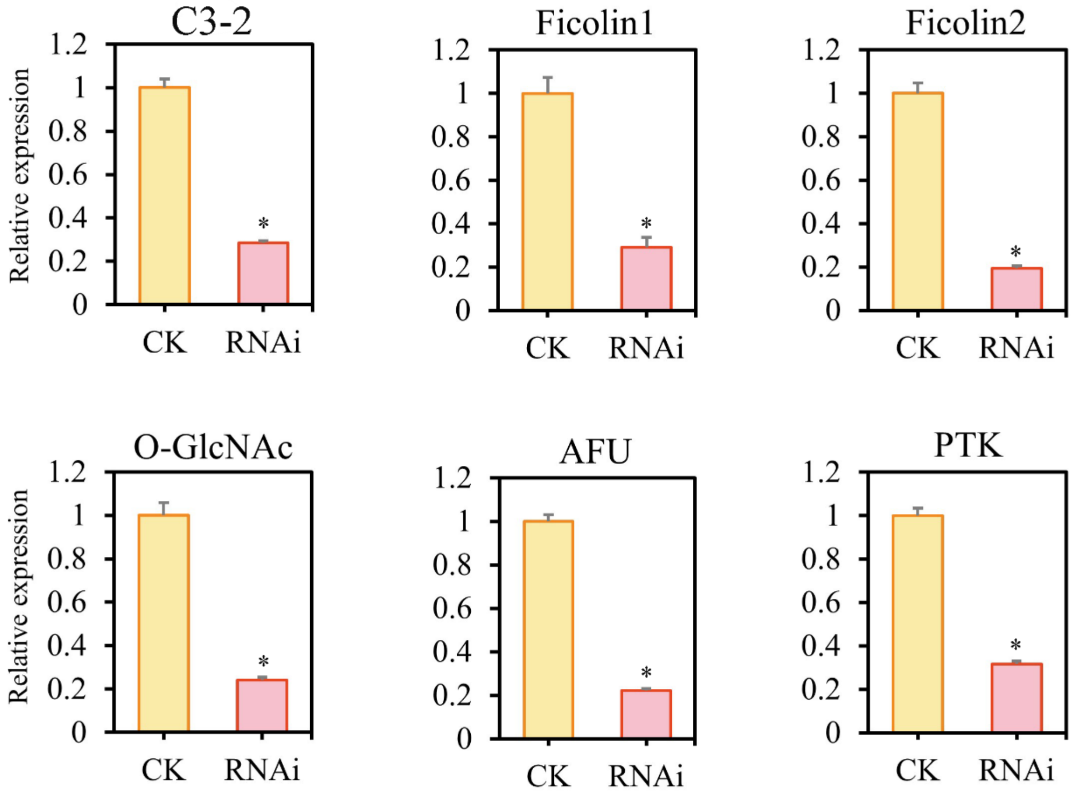

Figure S4. Relative expression levels after knockdowns of the identified factors; ∗p<0.05, significantly different as compared with the control group (CK, sterile seawater injection); RNAi, the RNA interference group (siRNA injection).

Table S1 The primer sequences for RACE used in this study

Primer name	Sequence (5′–3′)	Sequence source
AjAACT-1-3I	TGTAGTTGCTTGGGCACAGGCAGGAGTA	Designed in this study
AjAACT-1-3O	GAGGAGTGAGTTCAGGTTTGGGGACAGC	Designed in this study
AjAACT-1-5I	TGCTGTAAGTACCTGACCCAAAATAACC	Designed in this study
AjAACT-1-5O	TGGTGTCAGCCCTCTTTTGTCTGCCTCT	Designed in this study
AjAACT-2-3I	ATTAGCCAACATCAAACTCCTCGGTCTC	Designed in this study
AjAACT-2-3O	AATGTGGAGCCGTTAGCCAGGGTTGTCA	Designed in this study
AjAACT-2-5I	CCCTGGCTAACGGCTCCACATTCAACCT	Designed in this study
AjAACT-2-5O	AGCAATGGGGAACTCTATGGGAGCGACA	Designed in this study
AjHMGS-3O	GCTATTCCCTGGTACTTGGTATCTCACG	Designed in this study
AjHMGS-5I	AGCCCCAAGGTAGCACTGGATGGAAAGT	Designed in this study
AjHMGR-3I	CTTGGTTGGTTCAGCAATGGCAGGTAGC	Designed in this study
AjHMGR-3O	ACATCTGTTCCTGAGGTTTCAATCGGTG	Designed in this study
AjHMGR-5I	TGGCGTCTCCTGTCACCGATTGAAACCT	Designed in this study
AjMVK-3I	AGATAATGCCGTCAGCGTGTCCG	Designed in this study
AjMVK-3O	TGGCGGCGGGACTTCTTCTAGT	Designed in this study
AjMVK-5I	CAGACTGGGCATTTCGTCCAACGGTTCA	Designed in this study
AjMVK-5O	TGTCTCTCACTCCAGCTACCAGCACCTT	Designed in this study
AjPMK-3I	AGAGGATTAGTGCCCACGAAAGGT	Designed in previous study [28]
AjPMK-3O	AGGAGCAGCACAGGGCAGCTATGA	Designed in previous study [28]
AjPMK-5I	TTGTCGAGTCCACACTCTGACGGAGCAT	Designed in previous study [28]
AjPMK-5O	CCCACACCTTCTCTGTCCTCTCTGCCAT	Designed in previous study [28]
AjMVD-3I	AGGTTTGCCATCGGAGAGTAACCA	Designed in this study
AjMVD-3O	CTCCTGGTCTATCGTGAGGCTCGT	Designed in this study

AjMVD-5I	GGTCATCTCTTGAGCTCCAGGACCAACC	Designed in this study
AjMVD-5O	TCCCCTTCCCCCTCCGATATAGTCCACA	Designed in this study
AjIDI-3I	TCCCCTTCCCCCTCCGATATAGTCCACA	Designed in this study
AjIDI-3O	TCGGGTGAAACTGTTAGCTGACGAATGT	Designed in this study
AjIDI-5I	TCGGGTGAAACTGTTAGCTGACGAATGT	Designed in this study
AjIDI-5O	TGGGCTCTGGATTCACCATCACGTCTTT	Designed in this study
AjFPS-3I	CGTCCCACATGGAAAGCGAAACAGAGGC	Designed in previous study [29]
AjFPS-3O	TGAGGAGGAGGCCAGTAAAGATGCTGCA	Designed in previous study [29]
AjFPS-5I	GCCTCTGTTTCGCTTTCCATGTGGGACG	Designed in previous study [29]
AjFPS-5O	AGCAGCTCCACACACCATCCTAACACCA	Designed in previous study [29]
AjSS-3I	TACCAAGAGCAACGAGAAAGATAAAGCC	Designed in this study
AjSS-3O	TCCTTCCATCCTCACGAACTCTACAATC	Designed in this study
AjSS-5	GAGCGGAACTTTCTCTTCGTTGGGAATGGT	Designed in this study
AjSE-3	ATCGCTGGTGGAAGGCATTGTGGGTAGAGT	Designed in this study
AjSE-5	CGTAAAACTCTCGTCAACACCAAACCGC	Designed in this study
